# Transcriptomics Integrated with Metabolomics Reveals 2-Methoxy-1, 4-Naphthoquinone-Based Carbon Dots Induced Molecular Shifts in *Penicillium italicum*

**DOI:** 10.3390/jof8050420

**Published:** 2022-04-20

**Authors:** Xiaodan Chen, Wei Li, Jianying Chen, Xiaoyong Zhang, Wei Zhang, Xuewu Duan, Bingfu Lei, Riming Huang

**Affiliations:** 1 Guangdong Provincial Key Laboratory of Food Quality and Safety, College of Food Science, South China Agricultural University, Guangzhou 510642, China; 18125907913@163.com; 2 Key Laboratory for Biobased Materials and Energy of Ministry of Education, College of Materials and Energy, South China Agricultural University, Guangzhou 510642, China; liwei@scau.edu.cn (W.L.); jianychan@stu.scau.edu.cn (J.C.); 3 Joint Laboratory of Guangdong Province and Hong Kong Region on Marine Bioresource Conservation and Exploitation, College of Marine Sciences, South China Agricultural University, Guangzhou 510642, China; zhangxiaoyong@scau.edu.cn; 4 State Key Laboratory of Quality Research in Chinese Medicines, Macau Institute for Applied Research in Medicine and Health, Macau University of Science and Technology, Taipa, Macau 999078, China; wzhang@must.edu.mo; 5 Guangdong Provincial Key Laboratory of Applied Botany, South China Botanical Garden, Chinese Academy of Sciences, Guangzhou 510650, China; xwduan@scbg.ac.cn

**Keywords:** *Penicillium italicum*, 2-methoxy-1, 4-naphthoquinone, MNQ-based carbon dots, transcriptome, metabolome, mechanism

## Abstract

*Penicillium italicum* (*P. italicum*), a citrus blue mold, is a pathogenic fungus that greatly affects the postharvest quality of citrus fruits with significant economic loss. Our previous research showed that 2-methoxy-1, 4-naphthoquinone (MNQ) inhibited the growth of *Penicillium italicum*. However, the water dispersibility of MNQ will limit its further application. Herein, we synthesized MNQ-based carbon dots (2−CDs) with better water dispersibility, which showed a potential inhibitory effect on *P. italicum* (MIC = 2.8 μg/mL) better than that of MNQ (MIC = 5.0 μg/mL). Transcriptomics integrated with metabolomics reveals a total of 601 differentially enriched genes and 270 differentially accumulated metabolites that are co-mapped as disruptive activity on the cell cytoskeleton, glycolysis, and histone methylation. Furthermore, transmission electron microscopy analysis showed normal appearances and intracellular septum of *P. italicum* after treatment. These findings contribute tofurther understanding of the possible molecular action of 2−CDs.

## 1. Introduction

*Penicillium italicum*, one of the pathogenic culprits of citrus fruit postharvest disease, can cause huge economic losses during picking, packaging, storage, transportation, and shelf life. In postharvest industrial processes, the fruit rot rate generally remains around 20% because of easily spreading and contaminating quantities of disease once the peel is mechanically damaged [1]. Moreover, fungi have strong adaptability to extreme environmental conditions, which makes food preservation difficult. Therefore, the quest for efficient antifungal agents has played an important role in past research. Researchers found synthetic fungicides, such as pyrimethanil, imazalil, fludioxonil, and tiabendazole. Evidence suggests that prolonged and widespread use of antifungal drugs has contributed to some long-lasting consequences for fungi in humankind and the environment, especially the emergence of antimicrobial resistance among fungi worldwide [2]. Hence, research interests in recent years have focused on the development of excellent antimicrobial efficacy, high biocompatibility, and feasible and low-cost natural antifungal agents [3].

Plant-derived drugs, such as edible plants extracts, volatile oil, and medicinal plant extracts, play a critical role in studies of novel antifungal agents [1]. Numerous studies on edible plant extracts have examined the antimicrobial activity of aloe gel extracts, extracts of garlic, and *Plantago lanceolata* extracts [4], which showed significant antifungal activity on *P. italicum*. Other researchers have turned their attention to volatile oil from plants, such as clove essential oil. For example, clove essential oil is proven to have a potential antifungal mechanism that may be related to the rise in H_2_O_2_ levels, the suppression of MDA accumulation, and the induction of built-in cellular defense-associated enzymes [5]. In addition, the development of medicinal plant extracts with antifungal effects is worthy of attention. In our previous investigation, 2-methoxy-1, 4-naphthoquinone (MNQ) isolated from *Impatiens balsamina* Linn, which is a Chinese medicinal plant, was found to significantly inhibit *P. italicum* by disrupting metabolic processes, especially in energy metabolism and stimulus–response, which are both critical for the growth of the fungus [3]. Otherwise, MNQ mainly inhibits the growth of *P. digitatum* by affecting the synthesis of branched-chain amino acids and cell walls [6]. Based on our previous research, 2-methoxy-1, 4-naphthoquinone (MNQ) is a promising option for the prevention of postharvest decay. However, on the one hand, its poor water solubility may impact the safety of subsequent consumption, which is highly likely to limit the application prospects of MNQ. On the other hand, the stability of MNQ isolated from natural plants has a great impact on its application. Recently, our team has been seeking an optimized modified protocol.

With recent advancements in nanoscience and nanotechnology, carbon dots (CDs or carbon quantum dots), a new composite nanomaterial, have gradually become a rising star in the nanocarbon family because of their good antibacterial properties [7] and high biocompatibility [8]. CDs are generally regarded as small carbon nanoparticles below 10 nm in size. Oxygen, hydrogen, and other low-toxicity elements may comprise such CDs [9]. Their superior properties, such as excellent biocompatibility, low cytotoxicity, high chemical stability, optical transparency, conductivity, high mechanical strength, and narrow bandgaps [10], highlight the potential of CDs in applications such as anti-counterfeiting [11,12], information encryption methods [12], antimicrobial agents [13], and treatment of Alzheimer’s disease [14]. Otherwise, the preparation of carbon dots has the advantages of low cost, mass production, and simple operation, especially with green synthesis. The functionality of CDs depends on the nature of the precursors [15]. Therefore, it is necessary to turn MNQ into MNQ-based CDs. In order to obtain more effective antifungal drugs with better water solubility, we used 2-methoxy-1, 4-naphthoquinone as the carbon source to synthesize CDs by one-pot hydrothermal treatment.

## 2. Materials and Methods

### 2.1. Synthesis of CDs

For 2−CDs and 5−CDs synthesis, 2-methoxy-1, 4-naphthoquinone was used as a carbon source, and dimethyl sulfoxide (DMSO) was utilized as a solvent. For 2−CDs, 2-methoxy-1, 4-naphthoquinone (20 mg), and 10 mL of DMSO were ultrasonicated for 10 min and then introduced to a 15 mL Teflon reactor. Next, the reactor was heated to 120 °C for 6 h in an oven. In the next step, the reaction system was cooled down to room temperature, and 2−CDs with clear yellow dispersion were obtained. Dried 2−CDs were obtained by lyophilizing (vacuum lyophilization) the resulting mixture. Likewise, 2-methoxy-1, 4-naphthoquinone (20 mg) and 10 mL of DMSO were ultrasonicated for 10 min and then introduced to a 15 mL Teflon reactor to obtain 5−CDs. Then, the temperature of the reaction mixture was reduced to 25 °C after being heated to 180 °C for 6 h. The frozen mixture was lyophilized to obtain 5−CDs with the same traits.

### 2.2. Microorganism and Minimum Inhibitory Concentration (MIC) Test

*P. italicum* used in our experiment was rendered by the South China Botanical Garden, Chinese Academy of Sciences (Guangzhou, China). Potato dextrose agar (PDA, potato, 200 g/L; agar, 20 g/L; and dextrose, 20 g/L) was used as a medium. 

Minimum inhibitory concentration (MIC) determines the antifungal effect of 2−CDs on *P. italicum*. The conidia on the PDA plate were washed by sterile water for a dilution of 1 × 10^7^ conidia/mL. Drops (10 μL) of each conidial suspension were homogeneously seeded on the surface of each 60 mm dish. A volume of 10 mL PDA treated with 2−CDs (dissolved in DMSO) was added to the Petri dish. The 2−CDs concentrations were 0.0, 0.7, 1.4, 2.8, 5.6, 11.2, 22.4, and 44.8 μg/mL. The 5−CDs concentrations were 0.0, 0.5, 1.0, 2.0, 4.0, 8.0, and 16.0 μg/mL. Finally, all dishes were cultured at 28 °C for 2 days. To test MIC in PDB liquid medium, the conidia on the PDA plate were washed with sterile water for a dilution of 1 × 10^7^ conidia/mL. A volume of 2 mL of conidial suspensions were homogeneously seeded to each conical flask (1000 mL) containing 200 mL of PDB. The final 2−CDs concentrations were 0.0, 0.7, 1.4, 2.8, 5.6, and 11.2 μg/mL. Then, all conical flasks were shaken at 140 rpm at 28 °C for 2 days. All experiments were performed in triplicate and repeated with five biological replicates.

### 2.3. Effect of 2−CDs on the Growth of P. italicum

The conidia on the PDA plate were washed with sterile water for a dilution of 1 × 10^7^ conidia/mL. A volume of 20 μL of conidial suspension was added onto each 60 mm plate (10 mL modified PDA with 0.0 μg/mL 2−CDs, 1.4 (1/2 MIC) μg/mL 2−CDs, 2.8 (MIC) μg/mL 2−CDs). The experiments were performed repeatedly with three biological replicates. All plates were cultured at 28 °C, and the diameter of *P. italicum* was measured every 24 h.

### 2.4. Scanning Electron Microscopic (SEM) Observation

SEM observation was carried out on a scanning electron microscope (Zeiss, Oberkochen, Germany) to observe the morphology of *P. italicum* mycelia. SEM samples were prepared as described previously [6]. About 1.0 g of mycelia was collected from both treated and control media after 2 days of incubation. After quickly washing with PBS, the mycelia were fixed with pre-cooling 2.5% (*v*/*v*) glutaraldehyde in PBS at 4 °C for 12 h for fixation. The glutaraldehyde was removed by PBS (0.1 M, pH 7.4) twice. Next, it was dehydrated with a gradient ethanol series (30%, 50%, 70%, 80%, 90%, and 100% *v*/*v*), diluting with ethanol for 10 min each time, followed by isoamyl acetate replacement for 20 min and gold coating after conventional critical point drying. Subsequently, the samples were observed by SEM operating at a 3000 × magnification.

### 2.5. Transmission Electron Microscopic (TEM) Observation

Samples were pre-fixed in 5% glutaraldehyde in phosphate-buffered saline (PBS) at 4 °C for 12 h to fixate. The mycelium was washed four times in PBS (0.1 M, pH 7.4) for 30 min. The samples were postfixed in 1% OsO_4_ (EM Sciences, Hatfield, PA, USA) at 4 °C for 12 h and rinsed three times with PBS (0.1 M, pH 7.4), followed by a gradient ethanol series (30%, 50%, 70%, 80%, 90%, and 100% *v*/*v*), diluting with ethanol for 10 min each time. The dehydrated samples were infiltrated by immersion in a 1:1 mixture of epoxy propane and epoxy resin for 24 h, then embedded in the pure epoxy medium for 24 h. Ultrathin sections were taken using an EM UC6 ultramicrotome (Leica, Germany). A Talos-L120C transmission electron microscope (FEI, CZ) was applied to observe the sections after staining with uranyl acetate (EM Sciences) and lead citrate (EM Sciences).

### 2.6. Transcriptome Profiling of P. italicum

We obtained total RNA from the frozen samples of three biological replicates with Trizol reagent (Invitrogen, Carlsbad, CA, USA) in both groups. RNA quality and concentration were assessed using a Nanophotometer spectrophotometer (Implen, Munich, Germany), a Qubit ^®^ 2.0 fluorometer (Life Technologies, Carlsbad, CA, USA), and an Agilent Bioanalyzer 2100 system. A total of 3 μg of RNA per sample was used for cDNA library preparation using an Illumina TrueSeq RNA library preparation kit. The library fragments were purified with an AMPure XP system (Beckman Coulter, Beverly, CA, USA) for cDNA fragments of 250~300 bp in length. Then, the fragments were enriched by polymerase chain reaction (PCR) amplification to construct cDNA libraries. The cDNA libraries were sequenced on the Illumina platform with 125 bp/150 bp paired-end reads after quantification on an Agilent Bioanalyzer 2100 system. Only clean reads trimmed from raw reads were kept for further downstream analysis. Hisat2 (v2.0.5) was applied for alignment of high-quality reads to the reference genome of *P. italicum* before FeatureCounts v1.5.0-p3 was employed to calculate the read numbers mapped to each gene. Subsequently, the value of fragments per kilobase of transcript sequence per million base pairs sequenced (FPKM) was calculated based on the length of the gene and the read count mapped for each gene. For comparison, the resulting *p* values were adjusted using Benjamini and Hochberg’s approach for controlling the false discovery rate. Finally, *p* value < 0.05 and |log2 foldchange| > 1 were set as the criteria used for the screening of differentially expressed genes (DEGs) [6].

### 2.7. Untargeted Metabolome Analyses of P. italicum

The frozen samples (6 biological replicates for every group) were slowly thawed at 4 °C, and then 1 ml of precooled acetonitrile/methanol solution (acetonitrile: methanol: water, 2:2:1, *v*/*v*) was added and vortexed. Then, the samples were sonicated in an ultrasonic bath at low temperature (for 30 min) and incubated for 15 min at −20 °C to precipitate proteins, followed by centrifugation at 4 °C for 20 min. The vacuum-dried supernatants were redissolved in 100 μL water containing 50% (*v*/*v*) acetonitrile solvent and vortexed. Subsequently, the supernatant was collected after centrifugation at 14,000× *g* for 5 min at 4 °C for ultra-high-performance liquid chromatographic (Agilent 1290 Infinity LC) and mass spectrometric (AB Sciex Triple TOF 5600/6600) analysis in both positive and negative ion modes. In addition, quality control (QC) samples (10 µL of each sample) were homogeneously inserted to determine the reproducibility of the instrumental measurements. Analysis of the resulting extracts by employing hydrophilic interaction chromatography via electrospray ionization coupled to a quadrupole time-of-flight mass spectrometer (Sciex TripleTOF 6600) was efficient and comprehensive. Metabolites were separated on a Waters ACQUIY UPLC BEH column (2.1 × 100 mm, 1.7 µm) for 12 min with a gradient acetonitrile series of ammonium acetate (25 mM) and ammonium hydroxide (25 mM) (0 to 95%, *v*/*v*) at a 0.3 mL/min flow rate.

XCMS, a freely available software, was used to process the resulting data after the raw data (wiff. scan files) were converted to MzXML files by ProteoWizard MSConvert. Metabolites identification was attempted based on the precise mass of the molecules (<25 ppm) before their secondary spectral pattern concerning the in-house database. The data after normalization were subjected to data analysis, including principal component analysis (PCA) and orthogonal partial least-squares discriminant analysis (OPLS-DA). The variable importance in the projection (VIP) value from the OPLS-DA model contributed to the classification of metabolites. Metabolites with a VIP value > 1 and a *p* value < 0.05 were regarded as different metabolites [6].

### 2.8. RTq−PCR Analysis

According to the presentation of samples performed as previously described in omics analysis, we collected mycelia from liquid medium for RNA isolation. Total RNA from D−C and D−2CDs (*P. italicum* grown in PDB) were extracted using TRNzol (Invitrogen) before reverse transcription was performed to synthesize the first-strand cDNA for RTq−PCR assays using a Prime Script^TM^RT reagent lit (TaKaRa (RR047A)). The primers were designed and are listed in Table S1. Fluorescence changes of SYBR Green (TaKaRa (RR820A)) were monitored automatically in each cycle, and the threshold cycle (Ct) over the background was calculated for each reaction. Expression changes of target genes were normalized by reference gene GAPDH [16]. Relative expression levels between samples were determined using the 2^−∆∆Ct^ analysis method. All experiments were performed repeatedly with three biological replicates. 

## 3. Results

### 3.1. Characteristics of MNQ-Based CDs

**Figure 1:**
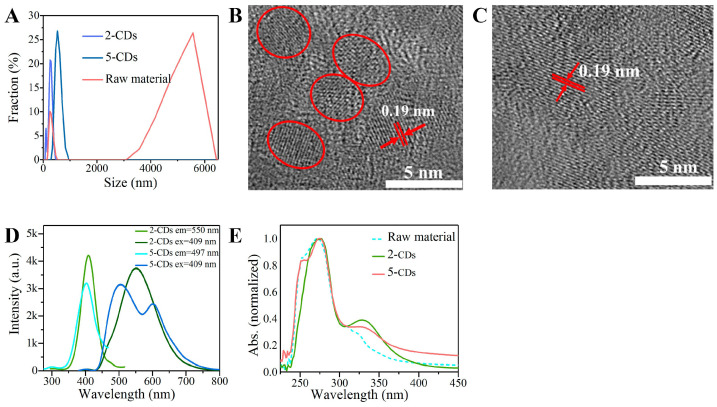
Appearance and optical characteristics of CDs. (**A**) Particle size distribution of CDs and raw material (2-methoxy-1, 4-naphthoquinone) in water. TEM images of 2−CDs (**B**) and 5−CDs (**C**). (**D**) Fluorescence excitation and fluorescence emission spectra of CDs. (**E**) UV–vis absorption of CDs.

Compared with the water dispersibility of raw materials, dynamic light scattering (DLS) measurements confirmed the smaller particle size of the CDs with a hydrodynamic diameter (Figure 1A), indicating that CDs feature better water dispersibility after carbonization. High-resolution transmission electron microscopic (HRTEM) images showed that the diameter of 2−CDs was about 4 nm, and the lattice fringe-spacing value was 0.19 nm (Figure 1B), corresponding to the characteristics of a graphite sp2 structure, indicating that 2−CDs contain graphite-like structures. As shown in Figure 1C, 5−CDs were uniformly dispersed, but a single particle was not obvious. Moreover, the lattice fringe-spacing value of 5−CDs was 0.19 nm and contained graphite-like structures. Compared with 2−CDs, 5−CDs featured lower water dispersibility.

To explore the fluorescence properties of CDs, ultraviolet-visible (UV–vis) absorption and fluorescence spectra were obtained. The UV-vis spectrum of the CDs (Figure 1E) showed good optical properties with a clear absorption peak. The 2−CDs and 5−CDs showed a strong absorption peak at 275 nm (Figure 1E). The fluorescence spectra showed a maximum excitation peak of 2−CDs and 5−CDs located at 409 nm, and CDs had a maximum fluorescence emission peak at 550 nm (Figure 1D).

**Figure 2:**
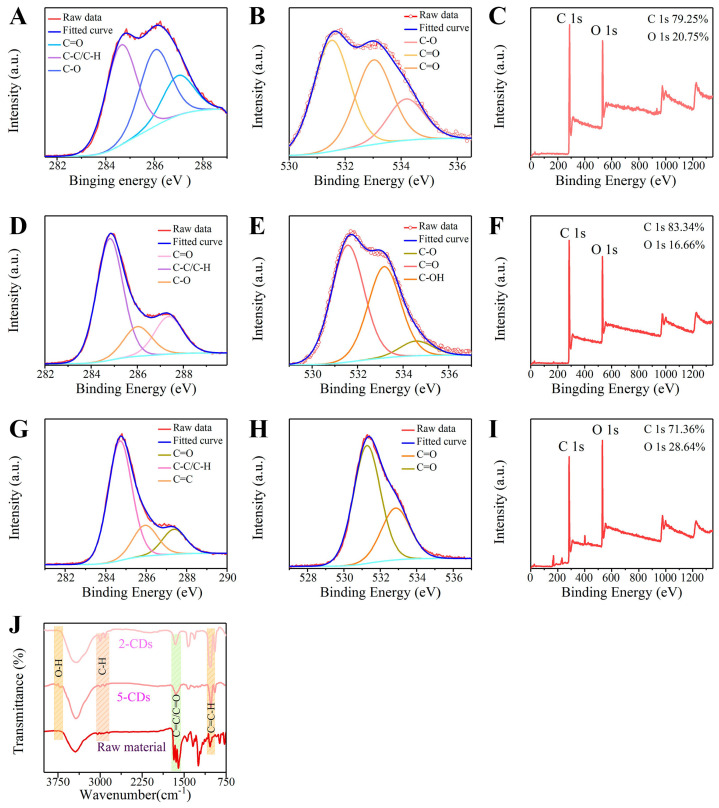
FTIR and XPS analysis. (**A**) Survey XPS spectrum of 2−CDs and the high-resolution XPS peaks of C1s (**B**) and O1s (**C**). (**D**) Survey XPS spectrum of 5−CDs and the high-resolution XPS peaks of C1s (**E**) and O1s (**F**). (**G**) Survey XPS spectrum of raw material (2-methoxy-1, 4 -naphthoquinone) and the high-resolution XPS peaks of C1s (**H**) and O1s (**I**). (**J**) FTIR spectra of 2−CDs, 5−CDs, and raw material (2-methoxy-1, 4-naphthoquinone).

Fourier transform infrared spectroscopy (FTIR) and X-ray photoelectron spectroscopy (XPS) were used to analyze the chemical composition of 2−CDs, 5−CDs, and the raw materials. FTIR showed that the surfaces of CDs all contain C-H (2918 cm^−1^, 2994 cm^−1^), C=O/C=C (1500–1700 cm^−1^), and CO-CH_2_ (1420–1430 cm^−1^) (Figure 2J). Stronger signals of C-H (2918 cm^−1^, 2994 cm^−1^) were detected in CDs. Meanwhile, the C-H (1650 cm^−1^) in the raw materials was totally from phenyl, which evidenced that the benzene ring might be decomposed by the heating reaction. In addition, the presence of an aromatic ring (865 cm^−1^) could be detected in the raw materials, but the signal of the aromatic ring was not detected in CDs, which certificated once again that heating promotes the decomposition of the benzene ring and the formation of CD characteristics. In addition, there was a strong infrared signal of -OH (860 cm^−1^) in 5−CDs, which indicates that the hydrophilicity of 5−CDs increases with the rising temperature and solvothermal reaction.

Survey XPS spectra revealed that C 1s, O 1s, and other constituent elements exist, with two corresponding peaks of 284 and 531eV. The atomic ratio of C 1s to O 1s of raw materials was 71.36% and 28.64%; the atomic ratio of C 1s to O 1s in 2−CDs was 79.25% and 20.75%; the atomic ratio of C 1s to O 1s in 5−CDs was 83.34% and 16.66%; the atomic ratio of C 1s to O 1s in 6-CDs was 88.35 and 11.65. By putting a perspective on the C/O atomic ratio of raw materials and CDs, we found that the carbon content and carbonization degree of CDs progressively increased with increasing carbonization temperature. This phenomenon might prove that the formation of CDs was accompanied by the carbonization and dehydration process. In the XPS high-resolution spectra of C and O of raw materials and CDs, CDs formed C-O bonds, which provided a strong proof of heating decomposing C=O of raw materials (2-methoxy-1, 4-naphthoquinone, MNQ). In addition, the spectral element and group analysis of X-ray photoelectron spectroscopy were highly consistent with the group analysis of Fourier transform infrared spectroscopy (Figure 2A–I).

### 3.2. Effects of 2−CDs and 5−CDs on P. italicum

**Figure 3:**
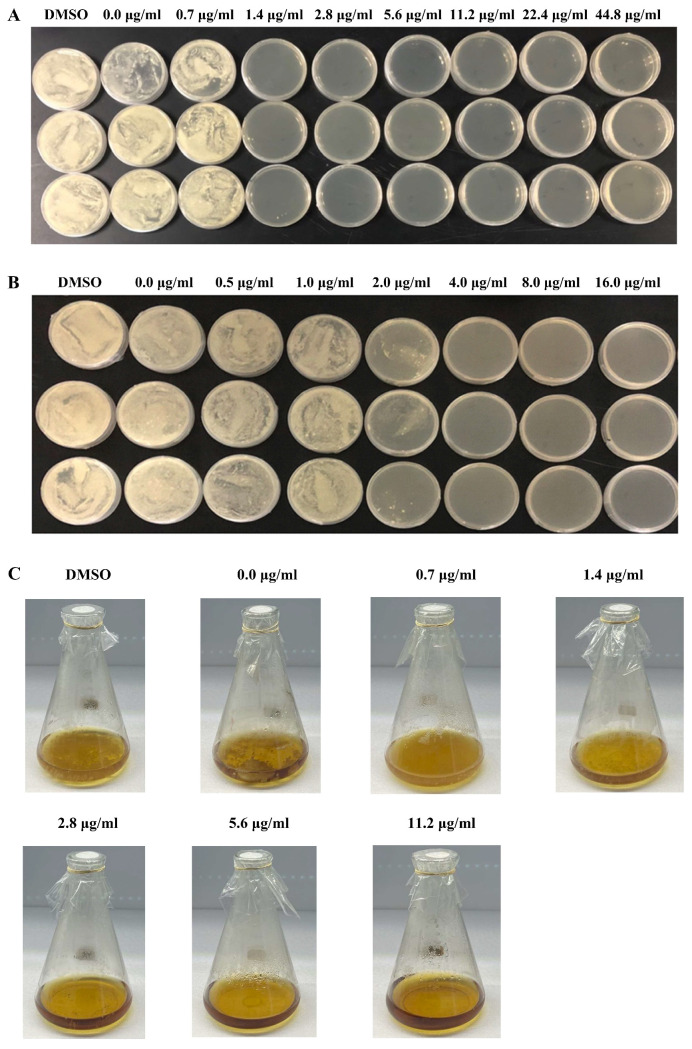
CD treatment on *P. italicum.* MIC of 2−CDs (**A**) and 5−CDs (**B**) against *P. italicum* (48 h) on PDA dishes; every dish line indicates one replicate; MIC of *P. italicum* after 2−CDs s treatment (48 h) in PDB liquid medium (**C**).

To explore whether CDs had more potent antimicrobial effects compared with MNQ, a preliminary pre-experiment was performed on *P. italicum*. Compared with MNQ, CDs inhibited pathogenic fungi more effectively (Figure S1). As shown in Figure 3A, after treatment with 2−CDs, *P. italicum* had impaired growth at 1.4 μg/mL and did not grow at all at 5.6 μg/mL. It is suggested that the MIC value of *P. italicum* treated with 2−CDs was 2.8 µg/mL. As presented in Figure 3B, *P. italicum* was inhibited by 5−CDs at an MIC value of 4.0 μg/mL. What emerged from the results reported here is that CDs showed a potential inhibitory effect on *P. italicum* better than that of MNQ (MIC = 5.0 μg/mL). Interestingly, 2−CDs had a more marked antifungal effect than that of 5−CDs (Figure 3A,B). In addition, the MIC of *P. italicum* in PDB liquid medium was consistent with that in PDA solid dishes (Figure 3C).

### 3.3. Effect of 2−CDs on the Growth and Morphology of P. italicum

**Figure 4:**
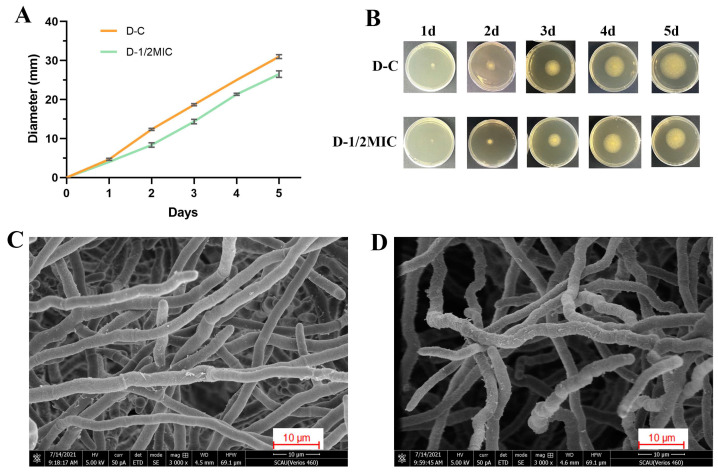
Effect of 2−CDs on the growth and morphology of *P. italicum*. (**A**,**B**) Effect of 2−CDs on the growth of *P. italicum*. SEM images of *P. italicum* mycelia under different cultivation conditions. (**C**) D−C: normal *P. italicum* of the control group (96 h); (**D**) D−2−CDs: *P. italicum* after treatment with 2.8 µg/mL 2−CDs (96 h).

As shown in Figure 4B, we observed the inhibitory effect of 2−CDs on *P. italicum*. After quantification, the growth curve showed a deceleration of the growth of *P. italicum* after 1/2 MIC treatment compared with D−C (Figure 4A). 

In addition, normal mycelium morphology is essential for the maintenance of fungal viability [17,18]. To observe the antifungal effect of 2−CDs on the mycelium morphology of *P. italicum*, morphological changes were visualized by scanning electron microscopy (SEM). Via the SEM images, it was revealed that the appearance of the control mycelia, which were compact, was regular and complete, and the surface of mycelia was smooth and flat without cracks (Figure 4A), whereas the mycelia treated with a 2.8 μg/L (MIC) concentration of 2−CDs showed considerable changes in their morphology. The mycelia with sparse distribution had a rough surface and were not as smooth as the normal group, accompanied by abnormal distortion (Figure 4B).

These SEM images suggested that there was a distinct morphological difference between the mycelia of D−2−CDs and D−C, which indicates that 2−CDs inhibited *P. italicum*. Therefore, the next section of research was concerned with the mechanism of 2−CDs against *P. italicum*.

### 3.4. Transcriptobomics Profile of P. italicum Treated with 2−CDs

**Figure 5:**
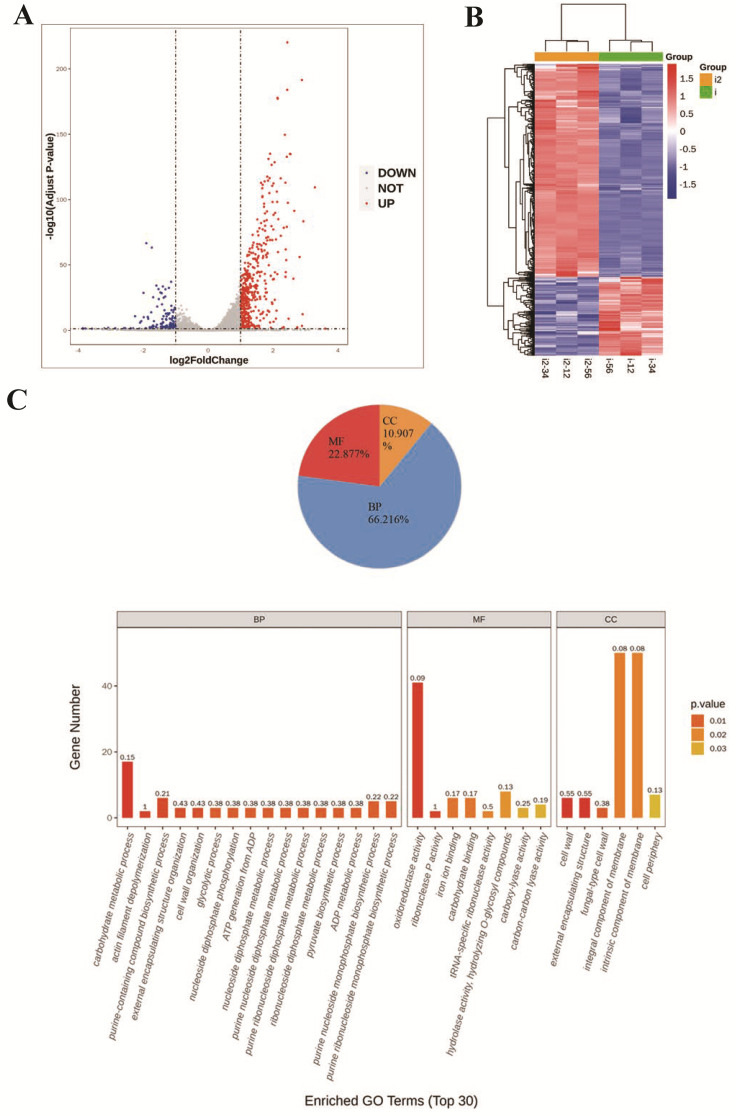

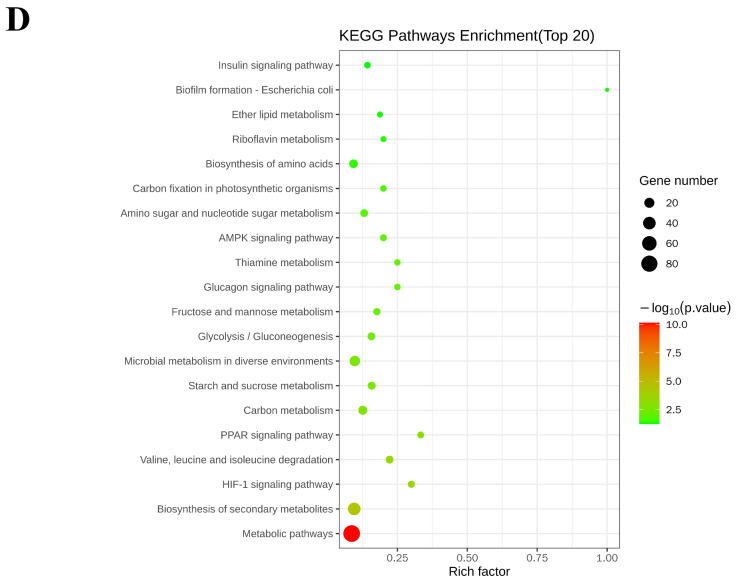
Transcriptobomics profile of *P. italicum* treated with 2−CDs. (**A**) Volcano plot of DEGs (|Log2 FC| > 1 and *p*-value < 0.05) between D−2−CDs and D−C. (**B**) Hierarchical cluster analysis of total DEGs (i: D−C, i2: D−2−CDs). (**C**) The top 30 enrichments of GO terms of DEGs. BP: biological processes; MF: molecular function; CC: cellular components. (**D**) Top 20 KEGG pathways of the identified DEGs. Red color: low *p*-value; green color: high *p*-value; circle size corresponds to gene number.

The transcriptomes of the mycelium samples made a great contribution to obtaining a global view of gene-expression changes between D−C and D−2−CDs [19]. There was a total of 9996 genes detected in D−2−CDs and D−C. What stood out in the volcano plot was the wide disparity between D−C and D−2−CDs, where a total of 601 DEGs was found (Table S2) (439 upregulated genes and 162 downregulated genes) (Figure 5A). All of the DEGs clustered into two groups, and the DEGs in the D−2−CDs did not cluster together with those in the D−C, which was revealed by hierarchical cluster analysis. (Figure 5B). Taken together, these analyses suggest that there is a significant difference in genes between D−C and D−2−CDs. Based on these results, we aimed to determine the functions of DEGs.

Gene ontology (GO) annotation offered a well-recognized method for the systematic functional analysis of target genes, which was applied to reveal the functions of DEGs [20,21,22]. The GO enrichment analysis of DEGs was divided into three functional categories, including biological processes (BP, 66.2%), molecular function (MF, 22.8%), and cellular components (CC, 10.9%) (Figure 5C). Most of the upregulated DEGs were enriched in BPs and MFs, whereas analysis of downregulated DEGs revealed that the vast majority of downregulated DEGs were also enriched in BPs and MFs, although they were more centrally located in BPs. The analysis of DEGs demonstrated that 2−CDs might have a strong ability to disturb both BPs and MFs. In BP categories, the most significant terms were “carbohydrate metabolic progress”, “actin filament depolymerization”, and “glycolytic progress”. In MF categories, catalytic activity genes (oxidoreductase activity, ribonuclease P activity, etc.) were mostly enriched (*p* < 0.05).

A total of 601 DEGs were detected between D−C and D−2−CDs, providing clues related to the underlying molecular mechanisms of 2−CDs against *P. italicum*. Subsequently, to identify the major functional pathways involved in the action of 2−CDs against *P. italicum*, both up- and downregulated DEGs were analyzed using Blast2go software and the KEGG database (http://www.genome.jp/kegg/) (accessed on 12 December 2021) [23], including 148 KEGG functional pathways (Table S3). The results of the KEGG pathway analysis indicated that there were 18 enriched pathways (*p* < 0.05). Interestingly, it was shown that most of the enriched functional pathways were related to metabolism, including metabolic pathways, biosynthesis of secondary metabolites, carbon metabolism, glycolysis/gluconeogenesis, starch and sucrose metabolism, etc. (Figure 5D).

### 3.5. Untargeted Metabolomics Profile of P. italicum Treated with 2−CDs

**Figure 6:**
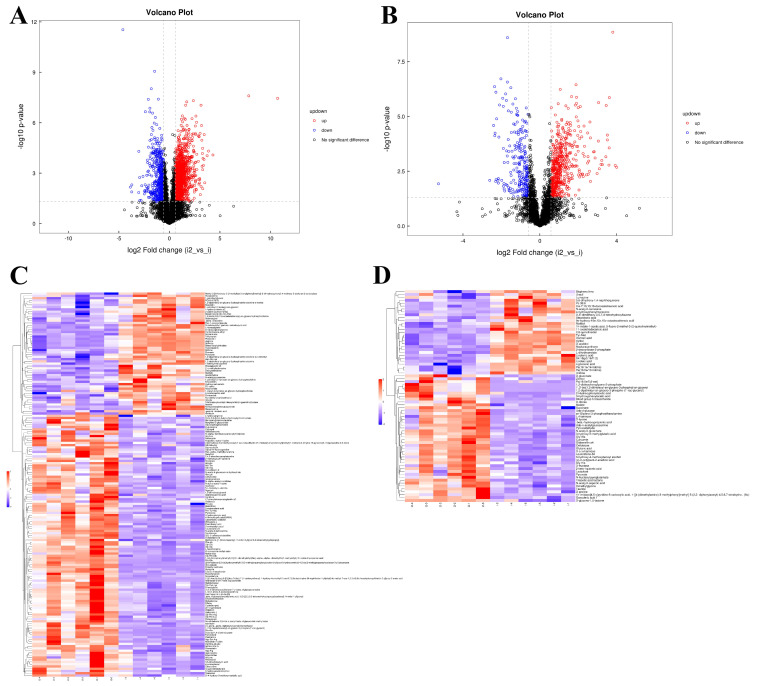

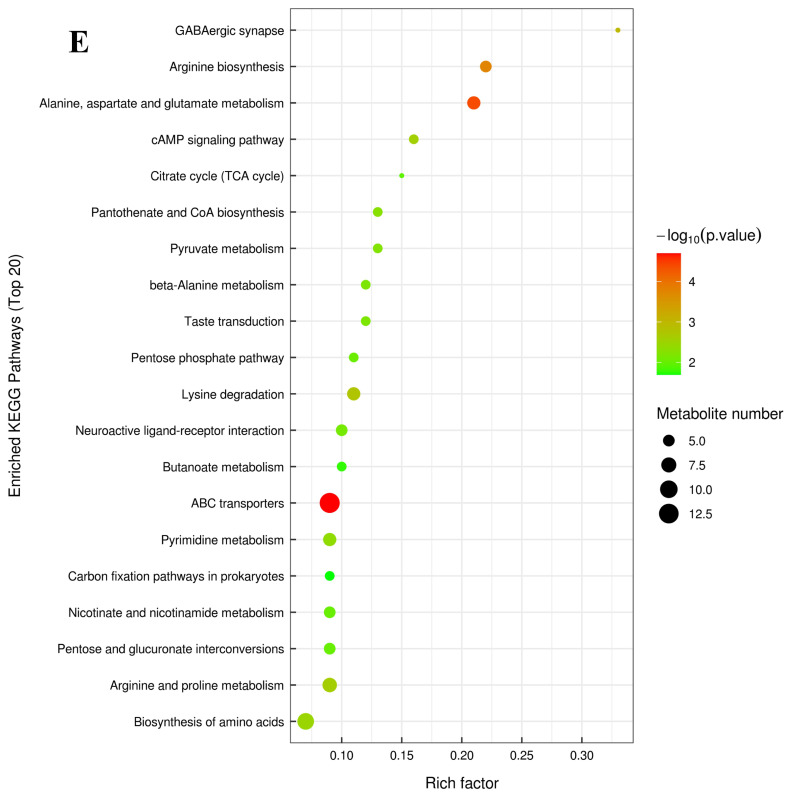
Untargeted metabolome profile of *P. italicum* treated with 2−CDs. Volcano plot of DAMs (differentially accumulated metabolites) in positive ion mode (**A**) and negative ion mode (**B**). Hierarchical cluster heat map of DAMs in positive ion mode (**C**) and negative ion mode (**D**) (i: D−C, i2: D−2−CDs). (**E**) Top 20 pathways encompassed by DAMs (VIP > 1, *p* value < 0.05). The rich factor represents the proportion of DAMs in the pathway among the metabolites detected. Color represents *p*-value; red color: low *p*-value, green color: high *p*-value; circle size corresponds to gene number.

To determine whether metabolites and metabolic pathways were changed after treatment, the samples of D−C and D−2−CDs were subjected to LC-MS untargeted metabolomics. The relative relationship between changes and metabolites by quantitative analyses of metabolites with low molecular weight could be revealed by metabolomics, which plays an important role in systems biology [24]. Extracted metabolites were analyzed in both positive and negative ion modes as described in Section 2.7. Based a VIP score greater than 1.0 (calculated using the OPLS-DA model) and a *p* value less than 0.05 (calculated by Student’s *t*-test), 270 metabolites were ultimately selected and considered as differentially accumulated metabolites (243 upregulated DAMs and 27 downregulated DAMs, Table S4). As shown in the volcano plot (Figure 6A,B), the metabolites were significantly different between D−C and D−2−CDs. Hierarchical cluster analyses displayed as thermograms indicated that the changes of within-group metabolites were not significant and that between-group changes were significant (Figure 6C,D), contributing to the study of changes in metabolic pathways and the accurate selection of target metabolites. 

Metabolic pathway analysis, an essential part of metabolomics research, helped to clarify the signaling pathways and metabolic pathways involved in metabolites; then, related metabolites and genes were explored [25]. KEGG enrichment analysis was performed on the differentiated metabolites, and 29 signal pathways were enriched (*p* < 0.05) (Table S5). As shown in the diagram, the top 20 pathways with lower *p*-values were selected to draw the KEGG enrichment histogram (Figure 6E). The pathways involved in amino acid metabolism and biosynthesis in these pathways were “alanine aspartate and glutamate metabolism”, “arginine biosynthesis”, “lysine degradation”, “arginine and proline metabolism”, “biosynthesis of amino acids”, “beta-alanine metabolism”, and “glycine, serine, and threonine metabolism”. We also found pathways involved in carbohydrate metabolism, such as “pyruvate metabolism”, “pentose phosphate pathway”, “pentose and glucuronate interconversions”, “citrate cycle (TCA cycle)”, and “butanoate metabolism”. Meanwhile, “carbon fixation pathways in prokaryotes” and “sulfur metabolism” are both involved in energy metabolism. Alternatively, the pathways involved in lipid metabolism were “linoleic acid metabolism” and “biosynthesis of unsaturated fatty acids”.

### 3.6. Transcriptomics–Metabolomics Integrative Analysis in P. italicum Treated with 2−CDs

**Figure 7:**
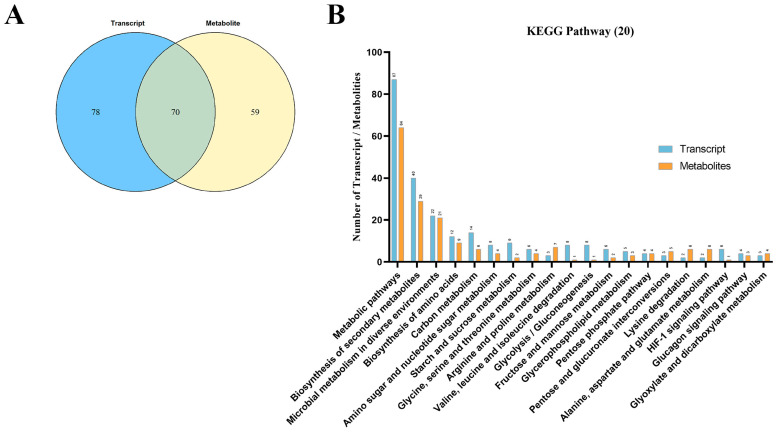
Transcriptomics–metabolomics integrative analysis in *P. italicum* treated with 2−CDs. (**A**) Venn diagram of the differentiated genes and metabolites. (**B**) Top 20 pathways with the largest number of DEGs and DAMs.

Combined transcriptome and metabolome analyses have been successfully employed to study the metabolic pathways of flavonoid biosynthesis in plants [26], stress resistance mechanisms [27], and the peel coloration of fruits [28]. Integrated transcriptomics–metabolomics analysis refers to the normalization of batch data from different biomolecular levels, such as transcriptome and metabolome, and statistical analysis to establish relationships between different levels of molecular data. It can not only clarify the changes in metabolic pathways related to DAMs but also analyze the corresponding DEGs. In addition, resulting transcriptomics and metabolomics data cam verify one another to reduce false positives resulting from single-omics analysis [29]. Therefore, conjoint analysis was necessary. We performed a correlation analysis of the transcriptome and metabolome using the Spearman statistical method in order to measure the degree of association between genes and metabolites in *P. italicum*, providing a new perspective. The matrix diagram was analyzed with R language and Cytoscape software, which not only showed the correlation between DAMs and DEGs but also showed correlations within each group (Figure S3). According to the changes in genes and metabolites, metabolic pathways were chosen as carriers, and a mapping analysis was performed to compare the data from the transcriptome and the metabolome. A total of 70 common target pathways between DEGs and DAMs were recognized (Figure 7A), and the top 20 pathways with the largest total number of DEGs and DAMs were enriched (Figure 7B). The most enriched pathways included “metabolic pathways”, “biosynthesis of secondary metabolites”, “biosynthesis of amino acids”, “carbon metabolism”, “starch and sucrose metabolism”, “glycine, serine, and threonine metabolism”, “arginine and proline metabolism”, etc.

## 4. Discussion

Results of a report of the World Health Organization (WHO) (https://www.who.int/dietphysicalactivity/fruit/en/index2.html) (accessed on 25 January 2022) suggest that vegetable and fruit consumption are often advocated as a component of healthy eating, which significantly increased fruit and vegetable consumption. In recent years, the postharvest high-quality fresh fruit industry has boomed, driven by increasing demand from consumers. What is frustrating is that citrus fruits, are extremely perishable products [30], such as *Citrus reticulate* Blanco, with a typical loose skin, and Chinese specialty citrus [31]. One of the main postharvest phytopathogens is *Penicillium italicum*, which is responsible for the blue mold disease.

Presently, fungicides such as pyrimethanil, imazalil, fludioxonil, and tiabendazole are widely used to control *P. italicum* [32]. However, the negative impacts of these toxic chemicals have caused public concern over human health and environmental risks [33]. Therefore, the use of such chemical antifungal drugs has faced increasingly more stringent regulation. In response to this growing problem, many new postharvest methods for control of citrus phytopathogens have been extensively explored, such as biocontrol methods, including antagonistic yeasts [34] and the application of essential oils [5]. Owing to their wide range of sources, pharmacological activities, low costs, and minimal side effects, natural products have attracted growing attention [1] for their potential to inhibit the growth of *P. italicum* [35]. 

Due to the excellent antifungal ability, good biocompatibility, and excellent water solubility of CDs, we used 2-methoxy-1, 4-naphthoquinone as a carbon source for 2−CDs and 5−CDs synthesis, and dimethyl sulfoxide (DMSO) was utilized as solvent. Compared with the raw material, the antifungal ability of MNQ-based CDs was improved, accompanied by greatly improved water solubility. This implies that only a small among of the drug remained on the surface after cleaning in later consumption. Because the antimicrobial ability of 2−CDs was stronger than that of 5−CDs, we chose 2−CDs as the material for the subsequent investigation of the antimicrobial mechanism. 

The normal morphology of the mycelium is very important for its physiological activity. In the present study, comparison of SEM images between two groups demonstrated the harmful effects of 2−CDs on *P. italicum*. Similar results were obtained in *P. italicum* treated with active extracts derived from poplar buds [36]. To further analyze possible mechanisms involved in the inhibition of *P. italicum* after 2−CDs treatment, we conducted a combined transcriptomic and metabolomic analysis (Tables S6 and S7). 

The cytoskeleton, a complex interacting meshwork that involves processes such as cell division [37], is mainly composed of actin, microtubules, and intermediate filaments in eukaryotic cells [38]. The cytoskeleton responds to stimulus signals with a quick, tightly regulated reorganization [39]. The hyphae of filamentous fungi are among the most extremely polarized cells, whereas the regulation of the cytoskeleton has a critical role in polarity establishment and maintenance. Actin binding of the ADF/cofilin protein family, as well as the actin remodeling proteins, could disrupt actin filaments with abnormal expression, which might tip the reorganization balance [40]. A relatively conserved role for the cytoskeleton of fungi showed a potential to open new avenues for antifungal treatments by disruption of the fungal cytoskeleton [41]. 

To probe whether an abnormal cytoskeleton would affect *P. italicum*, we first investigated the gene expression of cytoskeletal factors in the transcriptomic data. It was found that the expression levels of cofilin and CAMKK2, which are related to the actin filament depolymerization process, were significantly upregulated. This result indicates that the monomer dissociation at the negative end of the microfilament was accelerated. The broken dynamic equilibrium of dissociation and polymerization might be caused by the loss of the original function of the cytoskeleton. The cells with damaged cytoskeletons are unable to respond to external stimuli, thereby reducing resistance. The depolymerization of actin filaments requires a large amount of ADP, which could potentially break the dynamic equilibrium of ATP. 

According to omics analysis, 2−CDs could cause disorders in several metabolic pathways of *P. italicum*, especially intracellular metabolic pathways, including citrate cycle (TCA cycle) and glycolysis. As the central metabolic pathway in eukaryotic cells, glycolysis could provide energy and pyruvate to fuel the TCA cycle, as well as biosynthetic precursors for amino acids and secondary metabolite biosynthesis to cells. One interesting finding is that the expression of PFK1 and PGK as genes related to rate-limiting enzymes in glycolysis increased significantly. Correspondingly, pyruvate, a product related to glycolysis, was upregulated, which indicates that the glycolysis process was upregulated. Abnormal glycolysis aggravates the excessive consumption of carbon sources in a small area with an abnormal cytoskeleton, which might induce the detachment of glycolytic enzymes from the cytoskeleton [42]. Several enzymes related to glycolysis have been reported to be associated with the actin cytoskeleton [43,44]. Activated PFK1 can bond to the actin cytoskeleton and move along the cytoskeleton to initiate new glycolysis where needed. In addition to the changes in the cytoskeleton, PFK1 in the form of fructose-1, 6-bisphosphate performs a localized diffusion and is converted to adenosine triphosphate (ATP) along the growing cytoskeleton [45]. It was suggested that glycolysis is most likely organized around the actin cytoskeleton [46]. The detachment of glycolytic enzymes compartmentalizes glycolytic products, and the mass products cannot not be consumed in time locally, thus destroying the relatively stable internal environment that constitutes the cell to maintain normal life activities. 

In addition, the expression of pyruvate dehydrogenase (PDH), the key enzyme gene of the TCA cycle, an important pathway associated with energy metabolism [47], was inhibited. It was suggested that pyruvate might accumulate. The metabolomic data presented here provide experimental support for this assumption. The level of pyruvate significantly increased in D−2−CDs. To attempt to address this phenomenon, lactate dehydrogenase (LDH) might catalyze the conversion of pyruvate into lactate. We found that the concentration of lactate showed less obvious signs of increasing. Herein, it increased succinate levels, further suggesting the disruption of the TCA cycle. Together, this might indicate that 2−CDs interfered with intracellular ATP synthesis, which would affect the normal physiological activities of *P. italicum*. 

In eukaryotic cells, a highly ordered structure was defined as chromatin, composed of histones, DNA, and other chromosomal proteins [48]. Nucleosomes constitute the basic building block of chromatin, where each histone octamer consists of two copies: H2A, H2B, H3, and H4 [49,50]. Histones, a class of low-molecular-weight proteins rich in basic amino acids, are conservative in evolution. The tail of the core histone, which we call “histone tail” [51], is the main region where covalent modification occurs. Covalent modification of histones is critical for the regulation of gene expression. The methylation of the H3K4 site is among the most studied instance histone methylation and is mostly related to the active state of transcription. It has been reported that the transcriptional status of a genomic region is defined by H3K4me, which can prevent genome instability. Based on GO analysis, we found that there were several BPs related to histone methylation, such as histone H3K4 methylation, peptidyl-lysine methylation, histone lysine methylation, and histone methyltransferase complex. These BPs show a downward trend. In metabolomics analysis, pathways such as lysine degradation and arginine metabolism, as well the level of unmethylated histone, showed an upregulated trend, which confirms the above explanation. Therefore, a reduction in H3K4 methylation might reduce the transcription level of genes or invalidate the transcription. The low expression of the tRNA-specific ribonuclease-related gene indicates that there may be problems in the synthesis of tRNA. These conjectures suggest that amino acids might not be able to combine with tRNA and transport to ribosomes to synthesize proteins, which would cause the accumulation of a large number of amino acids, such as leucine and histidine. Hence, the abnormal translation process probably affected the normal physiological activities of *P. italicum* and possibly prevented the execution of the resistance signals of fungi.

**Figure 8:**
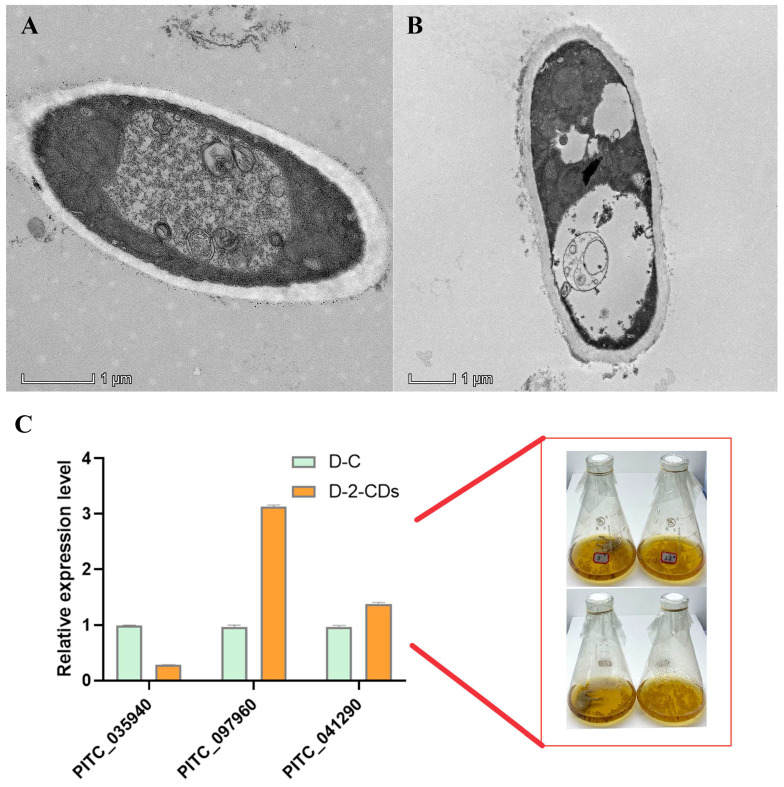
TEM images and RTq-PCR analysis for *P. italicum*. TEM observation: (**A**) D−C, (**B**) D−2−CDs; (**C**) relative expression level between D−C and D−2−CDs; D−C: *P. italicum* with no 2−CDs treatment (96 h); D−2−CDs: *P. italicum* treated with 2.8 μg/mL 2−CDs (48 h for routine culture and 48 h for 2−CDs treatment).

To verify the above assumption, we examined the intracellular structure of *P. italicum* in D−C and D−2−CDs. The *P. italicum* mycelia from D−C presented a typical fungal ultrastructure. In the D−C, all of the organelles, including the nucleus, the cell membrane, and the mitochondria, had normal appearances and intracellular septa with a dense cytoplasm that adhered to the plasma membrane and the cell wall. The *P. italicum* mycelia from D−2−CDs were not round and full. We observed indistinct intracellular organelles, altered cell wall thickness, and abnormal chromatin in *P. italicum*, owing to a dysfunctional cytoskeleton and abnormal histone methylation (Figure 8A,B). To further verify the result of omics analysis, the expression of some targeted genes of *P. italicum* was performed by RTq−PCR. The tested genes (PITC_097960, PITC_041290) were upregulated, and PITC_035940 was downregulated, which is consistent with the above assumption. This evidences strongly supports the notion that 2−CDs has an excellent inhibitory effect on *P. italicum*. 

## 5. Conclusions

Our data show that 2−CDs had a potential inhibitory effect on *P. italicum* (MIC = 2.8 μg/mL) better than that of MNQ (MIC = 5.0 μg/mL). Omics analysis revealed that a total of 601 differentially enriched genes and 270 differentially accumulated metabolites that are co-mapped as disruptive activity on the cell cytoskeleton, glycolysis, and histone methylation. Furthermore, TEM images and RTq−PCR verified the assumption. These findings contribute to further understanding of the possible molecular action of 2−CDs and provide some new targets for studies on antifungal agents.

## Data Availability

Not applicable.

## Supplementary Materials

The following supporting information can be downloaded at: https://www.mdpi.com/article/10.3390/jof8050420/s1, Figure S1: Preliminary test on *P. italicum* among 2−CDs, 5−CDs, and MNQ; Figure S2: PCA and OPLS-DA of metabolomics samples; Figure S3: Matrix diagram of the DEGs and DAMs; Table S1: Primers designed for RTq-PCR analysis of *P. italicum*; Table S2: Contents of DEGs; Table S3: KEGG analysis of the DEGs of *P. italicum*; Table S4: Contents of DAMs; Table S5: KEGG analysis of the DAMs in *P. italicum*; Table S6: Contents of the enriched GO terms of DEGs; Table S7: KEGG analysis of transcriptomics–metabolomics integrative analysis.
