# Supplementary material for: Transcriptomics Integrated with Metabolomics Reveals 2-Methoxy-1, 4-Naphthoquinone-Based Carbon Dots Induced Molecular Shifts in Penicillium italicum
Source: J Fungi (Basel). 2022 Apr 20;8(5):420. doi: 10.3390/jof8050420 (PMC9145997; doi:10.3390/jof8050420)
Supplement: Supplementary file 1 [file jof-08-00420-s001.zip › New Table S1-7/Table S1.pdf]

Table S1 Primers designed for the RTq-PCR analysis of *P. italicum*

Gene	Forward Primer	Reverse Primer	Length
PITC_041290	5′-GCGTCGGCGGAGAACTTAT-3′	5′-AGCAAGATTGTCGGCGAGAT-3′	~101bp
PITC_035940	5′-CTCCTCCTACAGGCCGAGAA-3′	5′-CTCCGACACCTTCGACAGCT-3′	~102bp
PITC_097960	5′-TCGCTGCCCAAGGAGTATTC-3′	5′-CCAAGCTCGTGCAACGTATG-3′	~102bp
PITC_050110	5′-TCCCGCAGATGGAAGGTTAA-3′	5′-TCGCTGTCAACGACCCTTTC-3′	~96bp
