# Supplementary figures and images for: Transcriptomics Integrated with Metabolomics Reveals 2-Methoxy-1, 4-Naphthoquinone-Based Carbon Dots Induced Molecular Shifts in Penicillium italicum
Source: J Fungi (Basel). 2022 Apr 20;8(5):420. doi: 10.3390/jof8050420 (PMC9145997; doi:10.3390/jof8050420)

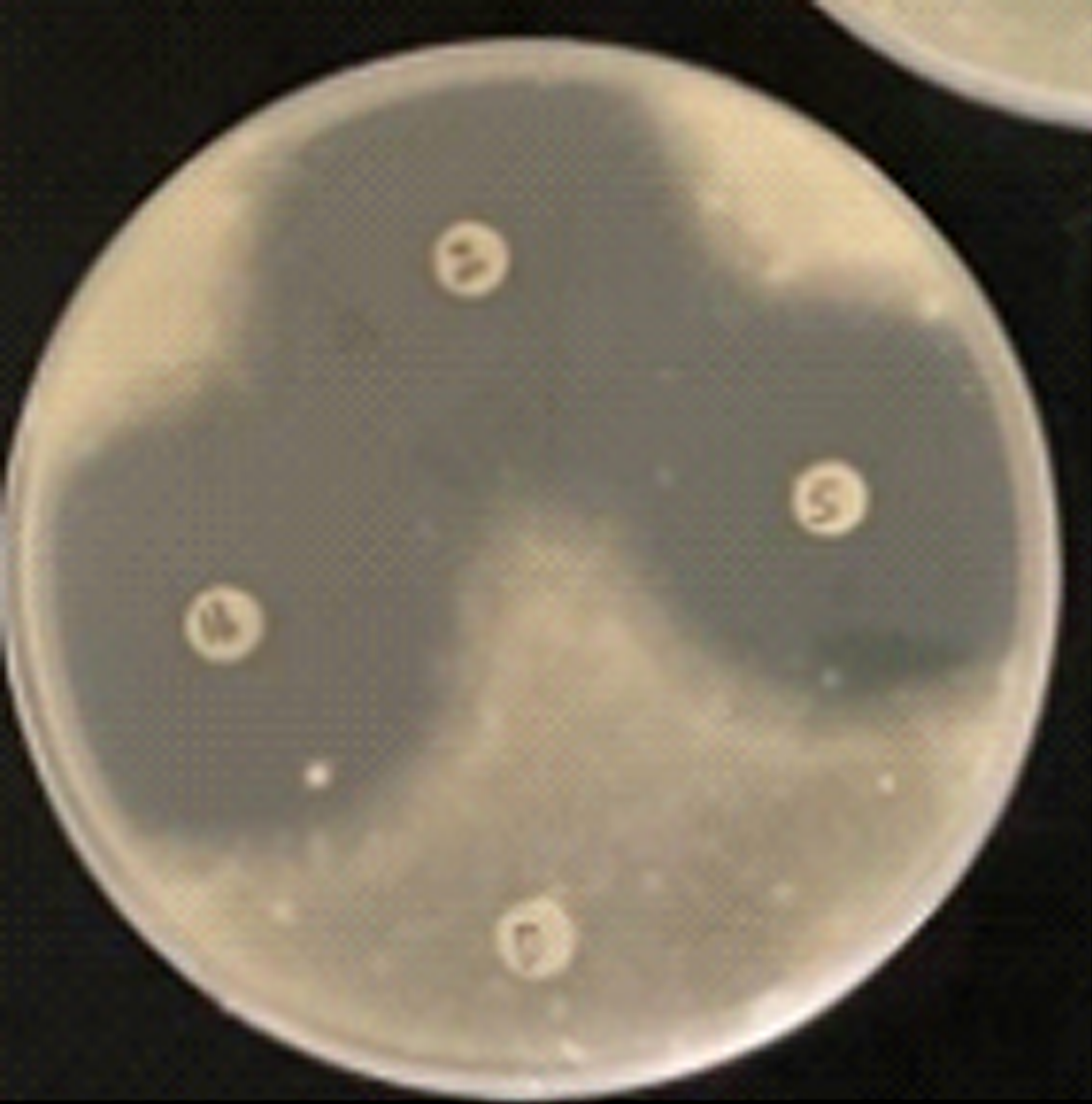

Supplement: Supplementary file 1 [file jof-08-00420-s001.zip › Figure S1-3/Figure S1.tif]

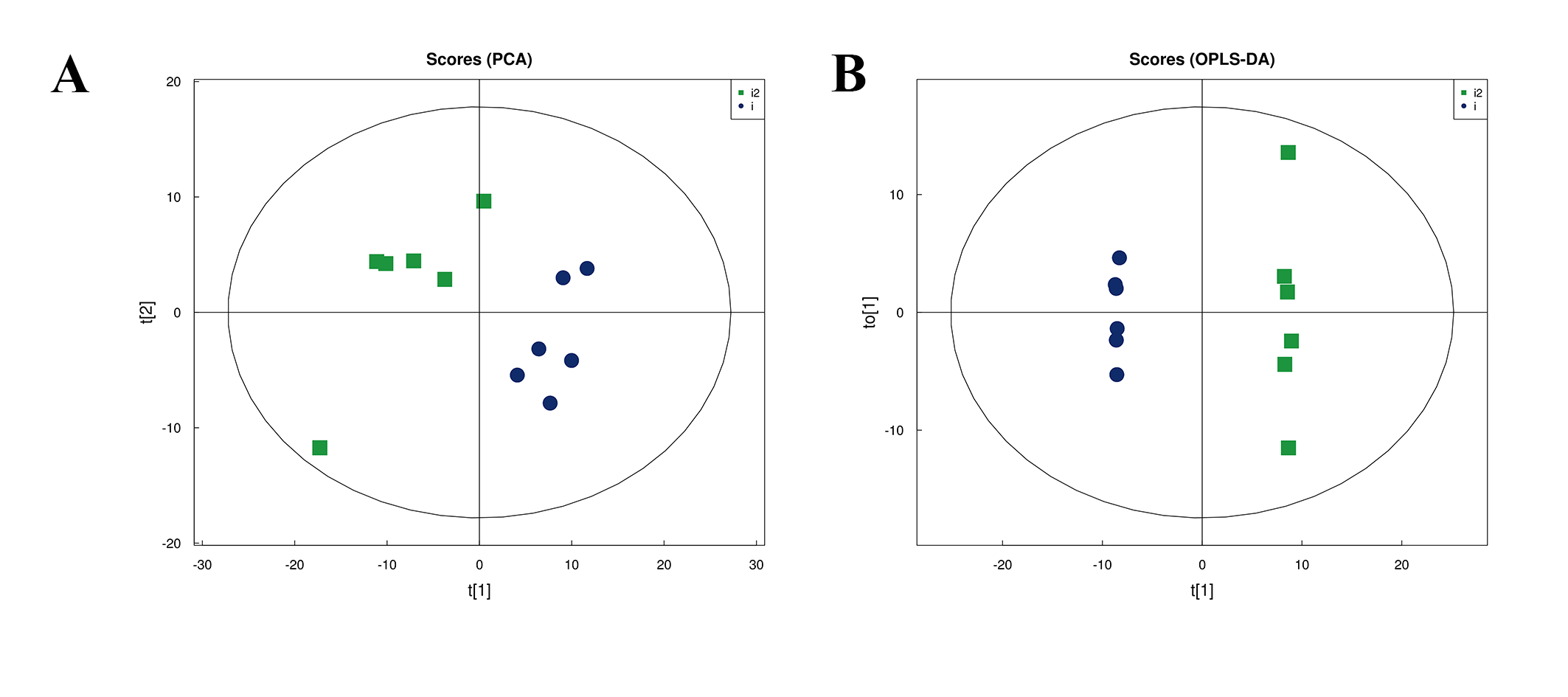

Supplement: Supplementary file 1 [file jof-08-00420-s001.zip › Figure S1-3/Figure S2.tif]

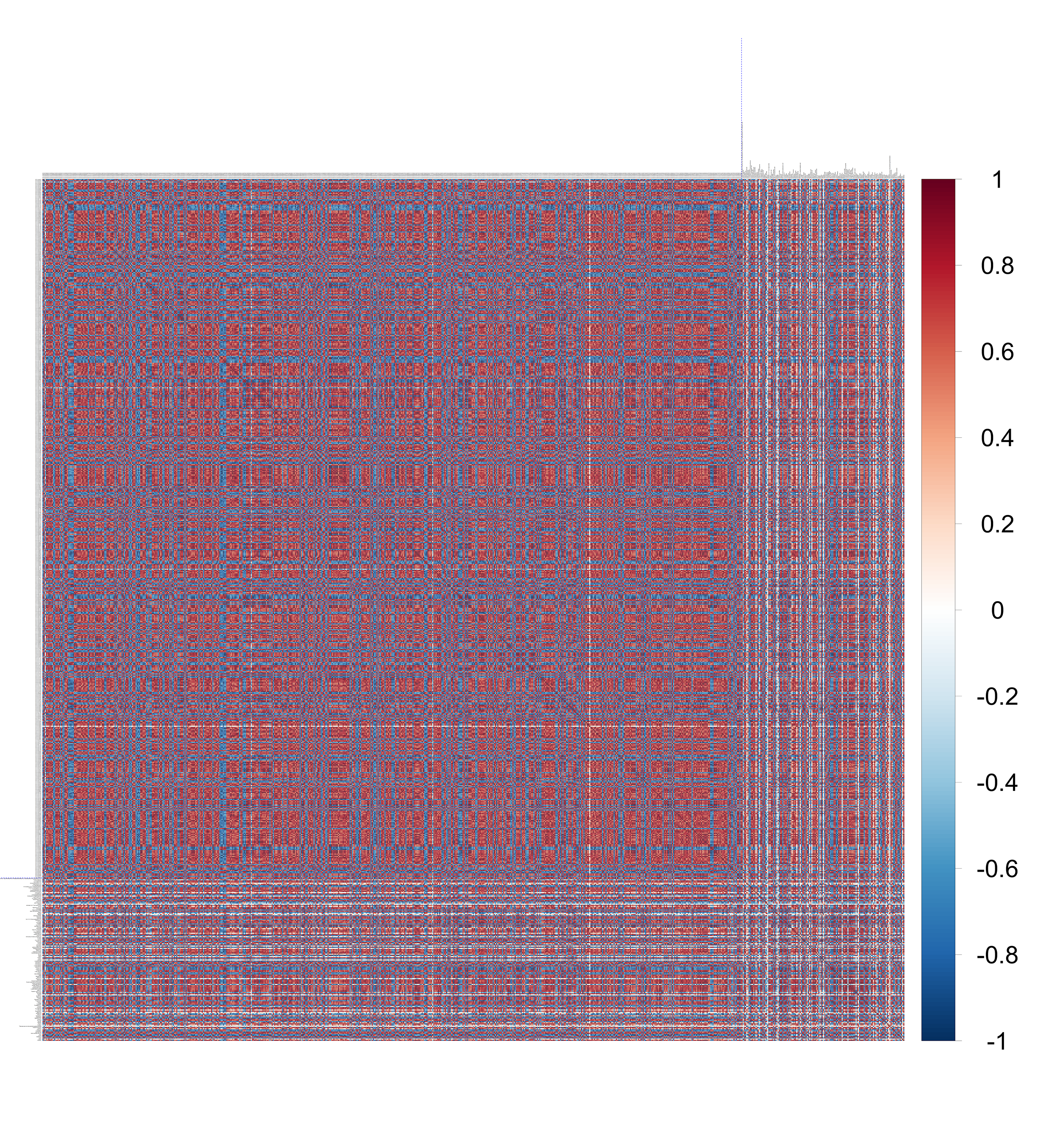

Supplement: Supplementary file 1 [file jof-08-00420-s001.zip › Figure S1-3/Figure S3.tif]
